# Ultrafast 3D spin-echo acquisition improves Gadolinium-enhanced MRI signal contrast enhancement

**DOI:** 10.1038/srep05061

**Published:** 2014-05-27

**Authors:** S. H. Han, F. H. Cho, Y. K. Song, J. Paulsen, Y. Q. Song, Y. R. Kim, J. K. Kim, G. Cho, H. Cho

**Affiliations:** 1 Department of Biomedical Engineering, UNIST, Ulsan, South Korea; 2 Schlumberger Doll Research Center, Cambridge, MA, USA; 3 Martinos Center for Biomedical Imaging, Massachusetts General Hospital, Charlestown, Massachusetts, USA; 4 Department of Radiology, Research Institute of Radiology, Asan Medical Center, University of Ulsan College of Medicine, Seoul, South Korea; 5 Korea Basic Science Institute, Ochang, South Korea

## Abstract

Long scan times of 3D volumetric MR acquisitions usually necessitate ultrafast *in vivo* gradient-echo acquisitions, which are intrinsically susceptible to magnetic field inhomogeneities. This is especially problematic for contrast-enhanced (CE)-MRI applications, where non-negligible T_2_* effect of contrast agent deteriorates the positive signal contrast and limits the available range of MR acquisition parameters and injection doses. To overcome these shortcomings without degrading temporal resolution, ultrafast spin-echo acquisitions were implemented. Specifically, a multiplicative acceleration factor from multiple spin echoes (×32) and compressed sensing (CS) sampling (×8) allowed highly-accelerated 3D Multiple-Modulation-Multiple-Echo (MMME) acquisition. At the same time, the CE-MRI of kidney with Gd-DOTA showed significantly improved signal enhancement for CS-MMME acquisitions (×7) over that of corresponding FLASH acquisitions (×2). Increased positive contrast enhancement and highly accelerated acquisition of extended volume with reduced RF irradiations will be beneficial for oncological and nephrological applications, in which the accurate *in vivo* 3D quantification of contrast agent concentration is necessary with high temporal resolution.

Acquisitions of 3D *T_1_*-weighted images with minimized susceptibility-induced decays benefit MRI scans, providing thin contiguous slices without cross-talks and unambiguous spatially specific positive contrast for *in vivo* applications with easier manipulation of resolution and signal to noise ratio (SNR)^1^,^2^,^3^,^4^,^5^,^6^,^7^,^8^. One of major drawbacks in acquiring such 3D volumetric images is the extended scan times from the multiple repetitions that are necessary to cover the increased number of phase-encodings for 3D *k*-space acquisition^9^. For this reason, ultrafast gradient-echo acquisitions, such as Echo Planar Imaging (EPI)^10^, or FLASH^11^,^12^ are widely used for 3D *T_1_*-weighted acquisitions. On the other hand, it is well known that gradient-echo images are intrinsically sensitive to susceptibility induced signal decays from magnetic-field inhomogeneities^10^,^11^,^12^. The sensitivity of gradient-echo readouts to susceptibility weighting is especially problematic for volumetric contrast-enhanced (CE) MRI with positive contrast agent, where non-negligible T_2_* effect of contrast agent tends to compromise the positive signal contrast and limits the range of MR acquisition parameters and injection doses for CE-MRI applications^13^,^14^,^15^. As a result, there are a large number of investigations on the development of novel positive contrast agent, focused in increasing the *in vivo* *r_1_*/r_2_* ratio to minimize susceptibility-induced decays, while maximizing positive signal enhancements^16^,^17^,^18^,^19^. However, only limited number of positive contrast agents is being clinically approved. In this work, we take an alternative way to maximize the positive signal enhancement of volumetric 3D CE-MRI using approved Gd-DOTA agent by implementing a new ultrafast 3D *T_1_*-weighted MR imaging method with spin-echo readouts.

Previously, for the reduction of susceptibility-induced decays and the accelerated acquisition, rapid spin-echo-train acquisition has been typically achieved using a Carr-Purcell-Meiboom-Gill (CPMG)-based, fast spin echo (FSE) sequence^20^,^21^, in which rapid scans are performed using spin-echo-trains from refocusing RF pulses. On the other hand, as one RF pulse is associated with one or two spin-echoes for CPMG-based fast spin echo sequences^20^,^21^, high RF power deposition may be problematic for repetitive 3D applications, in addition to the longer scan time compared with corresponding gradient echo acquisitions. GRASE sequence mixes gradient and spin echoes by using bipolar gradient readouts for each echo in FSE sequence to further reduce RF irradiations and scan time, but is inherently T_2_* weighted^22^. In this work, following the advantageous concept of previous spin-echo-train acquisitions, we demonstrate *in vivo* feasibility of increasing the positive signal enhancement of ultrafast 3D *T_1_*-weighted images for CE-MRI, by maximizing ratio of effective spin-echoes per each RF pulse and minimizing susceptibility induced decays with pure spin-echo readouts at the speed comparable to that of Echo Planar Imaging (EPI).

To increase the ratio of spin-echoes to RF pulses per repetition, several techniques have been developed by splitting the position of echo formation from the multiple coherence pathways generated by a relatively small number of RF pulses^23^,^24^,^25^,^26^,^27^,^28^,^29^,^30^,^31^,^32^. The Multiple-Modulation-Multiple-Echoe (MMME) sequence^33^,^34^,^35^,^36^, which maximizes the number of echoes (57) for 5 RF pulses ([54°–71°–71°–71°–110°]) within 30 ms ~ 50 ms of the total echo train time, provides an unique opportunity to reduce the necessary repetitions and RF irradiations with minimized susceptibility-induced decays during the spin-echo train. While in theory the number of echoes can be exponentially increased by adding RF pulses to further accelerate the scanning^33^, the later echoes from the MMME sequence tend to have a lower signal-to-noise (SNR) mainly due to the signal decay by diffusion. Such lack of high-SNR echoes in the latter parts of the MMME sequence with 5 RF pulses decreases the methodological efficiency to effectively fulfill the necessary repetitions for the entire 3D *k*-space coverage of high-resolution images. However, overcoming this shortcoming, recent technical advances in sparse sampling and reconstruction techniques have improved the 3D *k*-space coverage by increasing the effective phase encoding steps for each echo train of the MMME sequence.

A compressed-sensing-assisted MMME sequence (CS-MMME) was implemented in this work to further increase the ratio of spin-echoes to RF pulses per repetition and to significantly reduce both the susceptibility decays and the number of RF irradiations. Compressed sensing (CS) theory was recently established and proved that only a small fraction of the samples necessary for a regular linear reconstruction (i.e., fast Fourier transformation, Hadamard) is sufficient to reconstruct sparse or compressible signals given certain restrictions on the sampling^37^,^38^,^39^,^40^. MR images have long been known to be sparse under various spatial transformations, such as discrete wavelet transforms^41^,^42^,^43^. Furthermore, the ability to easily manipulate the sampling in a conjugate space of the image, i.e., *k*-space, makes it straightforward to obey the sampling requirements. Accordingly, following CS theory, 3D MR images can be reconstructed by solving an *l*_1_-norm minimization problem, i.e., minimizing the *l*_1_-norm of the compressed image that is consistent with the acquired data^44^,^45^. For the CS application for the MMME echo train sequence, multiple spin echoes from the MMME sequence were used to rapidly fill Cartesian 3D *k*-space lines for each echo train. In addition, the CS-assisted *k*-space sampling allowed the allocation of high-SNR echoes for the sparse sampling of phase-encoding lines. A multiplicative acceleration factor from multiple spin echoes (×32) and sparse sampling (×4–×8) was investigated for ultrafast acquisition of 3D *T_1_*-weighted images in multiple subjects, including susceptibility/relaxivity phantoms, fruits with fine morphology, and *in-vivo* animals over both short and long repetition times. First, we confirmed the reduced susceptibility artifacts of the MMME sequence over conventional EPI acquisitions with the glass-water phantom. Second, the proton-density contrast with minimized T_2_*- and diffusion weightings of non-slice-selective 3D spin-echo-train (multi-echo factor = 32) was verified with the MMME sequence using the phantom which was made of water, oil, and Gd-doped water at long repetition times. Third, we performed a *T_1_* measurement with a variable repetition time (TR) with the MMME sequence using differently doped agarose samples with Gd to verify the *T_1_*-weighted contrast at short repetition times. Fourth, the image quality was characterized by varying the MMME echo numbers, echo times, and CS acceleration factors for multiple phantoms with both fine morphology and *in vivo* animals. Finally, a 3D CS-MMME sequence was implemented to show the increased signal contrast with the *in vivo* injection of Gd-DOTA at two doses of 0.3 mmol/kg and 0.1 mmol/kg for multiple repetition times in rats. Signal enhancements were compared with corresponding conventional FLASH images in kidney of rats.

The results demonstrate the feasibility of combining two independent acceleration factors to achieve up to a 128 ~ 256 fold reduction in the number of repetitions required for a 3D *T_1_*–weighted spin-echo-train image acquisition, with the in-plane resolution of 200 μm in an animal scanner. Susceptibility artifacts and T_2_* weightings can be minimized with fewer RF irradiations at the acquisition speed close to corresponding EPI sequence. However, this comes, at the cost of respective inhomogeneities and blurring artifacts from MMME multi-echo and CS accelerations, requiring future work for the further improvements.

## Methods

All studies were performed on a 4.7-T MRI system (Bruker BioSpin, Billerica, MA) using a 72-mm volume coil (Bruker) and gradient strength up to 38 G/cm. *In vivo* rat experiments were performed in accordance with protocols approved by the Institutional Animal Care and Use Committee of the Korea Basic Science Institute (KBSI-IACUC, Ochang, Korea).

### MMME imaging sequence and amplitude/phase corrections

**Figure 1:**
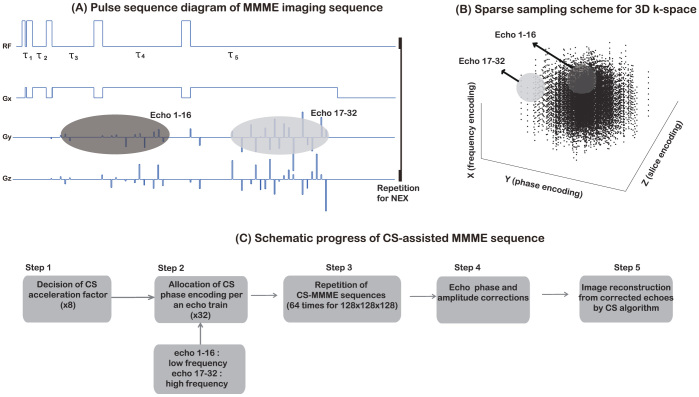
The pulse sequence (A), phase-encoding scheme (B), and schematic workflow (C) of CS-MMME sequence. Frequency encoding gradient is turned off during the RF pulses for non-slice-selective 3D acquisition in this work. The reference scan is performed by turning on only the frequency encoding gradient.

A schematic of the MMME sequence is shown in Figure 1A. A combination of five RF flip angles ([54°–71°–71°–71°–110°]) was used to reduce the echo amplitude variation in each echo spectrum^33^. The echo time (delay between the first and the second pulse) τ_1_ was determined by considering the digitization time and the size of the matrix, where τ_2_ = 3τ_1_, τ_3_ = 9τ_1_, and τ_4_ = 17τ_1_ were used to achieve the maximum separation of each echo.

The MMME imaging sequence is composed of a few RF pulses in the presence of a constant readout gradient, which separates the central position of each echo from the independent coherence pathway^33^,^34^. The maximum number of (3*^N^*^−1^ − 1)/2 echoes after *N* RF pulses can be generated using appropriately spaced RF pulses^33^,^34^. In principle, increasing the number of pulses increases the number of echoes and should lead to a higher-resolution image within a single echo train. However, molecular diffusion in the presence of the gradient attenuates the amplitude of each echo differently, leading to a lower-SNR image when the later low-quality echoes were included in image reconstruction. There are variations in the amplitude and phase of each echo resulting from the different coherent pathways of each echo^33^, leading to image artifacts unless proper correction steps are taken before Fourier reconstructions.

Thus, for fast-imaging applications using the MMME-generated echoes, two corrections need to be considered. For the amplitude corrections of the raw data, the amplitude ratio *w_i_* of the *i*th echo is calculated as *w_i_* = A*_m_*/A*_i_*, where A*_i_* is the maximum amplitude for each echo and A*_m_* is the largest amplitude among all the echoes from the reference scan without phase encoding gradients. Each raw echo is then multiplied by its weighting factor *w_i_*. For the phase corrections, the echoes from the reference scan were Fourier transformed in the frequency-encoding direction, and then, the relative phase of each echo was estimated. These phases were subtracted from the phase of the fully encoded echoes before performing Fourier transforms along the phase-encoding directions.

### Optimization of the compressed-sensing (CS) scheme for MMME

Compressed sensing (CS) can be described as reconstructing the signal from undersampled data by minimizing the *l*_1_-norm of the signal over the sparse domain, which is an operation known as the compressive transformation. This is achieved by solving the following constrained minimization problem^39^: 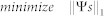

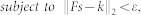
where the image that we are reconstructing is represented by the vector *s*, and *Ψ* denotes a sparse transformation operator, such as a wavelet transformation. The acquired undersampled *k*-space data is *k*, *F* is the Fourier transformation operator transforming the image into *k*-space, and *ε* controls not only the accuracy but also the speed of reconstruction. Usually, *ε* is set according to the noise level of the *k*-space data^46^. The reconstruction was performed using code developed in MATLAB (MathWorks, Natick, MA, USA) and two external packages: spgl1 v.1.7^47^ for solving the *l*_1_-norm minimization problem and Wavelab v.8.02^48^ for applying the wavelet (Symmlet) transformation. To implement the CS in the MMME sequence, the undersampling scheme was optimized. The undersampling of the phase encoding steps followed a random sampling scheme. The center *k*-space points in these schemes were always sampled, and the remaining points were sampled with Gaussian weighting. This scheme ensures a sampling bias towards small *k* values with a good SNR, while still collecting enough data for larger *k*-values to reconstruct the details of the image as well as maintaining sampling incoherence for CS. Because the frequency encoding cannot be undersampled, other two phase encoding steps were undersampled following the use of centered 2D Gaussian-shaped masks with acceleration factors of 4, 8, and 16 to cover 3D *k*-space. The phase-encoding scheme is shown in Figure 1B, where the black dots represent the actually sampled points for volumetric 3D MMME acquisition.

Figure 1C shows a flowchart of the acquisition and reconstruction scheme of the 3D CS-MMME sequence. For example, a 3D image with a matrix size of 128 × 128 × 128 (slice × phase × frequency) was chosen for reconstruction. After selecting 32 echoes from the MMME sequence using 5 RF pulses, a CS-acceleration factor of 8 was applied. Then the CS-selected phase/slice-encoding steps (128 × 128/8) were ordered according to total gradient strength (*G_y_*^2^ + *G_z_*^2^), and assigned to 32 echoes of the MMME sequence for each echo train. This process was repeated 64 times to fully cover the necessary encodings. In this way, we simultaneously acquired 256 (32 × 8) effective phase-encoding steps within a single echo train with 5 RF pulses.

### Phantom and in-vivo experiments with CS-MMME

First, we compared the MMME (multiecho × 32) sequences with conventional gradient-echo (GE)- and spin-echo (SE)- echo planar imaging (EPI) acquisitions using a susceptibility (glass tubes in a water) phantom to study the robustness of the MMME sequence to susceptibility artifacts. The imaging parameters for EPI were set as follows: TR = 3 s, NS = 1, FOV = 2.59 × 2.59 cm^2^, resolution = 405 × 405 μm^2^, slice = 1, and TE = 15 ms. Second, a FSE image at an ETL of 32 and a 3D MMME image at a corresponding multi-echo factor of ×32 were acquired with a relaxivity (water, oil, and Gd-doped water) phantom at long repetition times. The imaging parameters for the FSE sequence were set as follows: TR = 5 s, ETL = 32, NS = 1, FOV = 5 × 5 cm^2^, resolution = 195 × 195 μm^2^, echo spacing (ES) = 11 ms, effective echo time (TE _eff_) = 165 ms with linear *k*-space ordering. The imaging parameters of the MMME sequence were set as follows: TR = 5 s, NS = 1, FOV = 5.22 × 5.22 × 5.22 cm^3^, Matrix: 256 × 256 × 64, resolution = 203 × 203 × 813 μm^3^. The digitization time (Δt) was 5 μs, the phase-encoding gradient-on times for two directions (T_ph_, T_sl_) were 300 μs, the strengths of the unit phase-encoding blip along the phase encoding directions (ΔG_y_, ΔG_z_) were 0.15 G/cm, and the echo time (τ_1_) was 800 μs. For the verifications of *T_1_*-weighted acquisitions of the CS-MMME sequence, mixture samples of 0.05% agarose gel with Gd concentrations of 0.3 mM (*T_1_* = 542 ms), 0.5 mM (*T_1_* = 360 ms), 1 mM (*T_1_* = 213 ms) and 1.5 mM (*T_1_* = 152 ms) were imaged together with a variable-TR CS-MMME sequence. The values of TR were varied from 0.14 s to 1 s.

Third, a tangerine and a kiwi which has fine seeds were used for the image reconstructions to optimize the echo time, multi-echo factor, and CS factor for the CS-MMME sequence. The imaging parameters of the CS-MMME sequence were set as follows: TR = 3 s, NS = 1, FOV = 5.22 × 5.22 × 5.22 cm^3^, Matrix: 128 × 128 × 128(64) and 256 × 256 × 64, Δt = 5 μs, T_ph_ ( = T_sl_) = 150 μs and 300 μs, ΔG_y_ ( = ΔG_z_) = 0.3 G/cm and 0.15 G/cm, τ_1_ = 800 μs (800 μs) and 500 μs.

Fourth, an *in vivo* proton-density experiment was performed for further optimization. The imaging parameters of the proton-density CS-MMME sequence were set as follows: TR = 3 s, NS = 1, FOV = 5.22 × 5.22 × 5.22 cm^3^, Matrix: 256 × 256 × 64 and 128 × 128 × 64, Δt = 5 μs, T_ph_ ( = T_sl_) = 300 μs and 150 μs, ΔG_y_ ( = ΔG_z_) = 0.15 G/cm and 0.3 G/cm, τ_1_ = 500 μs and 500 μs, and CS factors at ×4, ×8, and ×16.

Finally, to demonstrate the direct benefit of the proposed sequence for CE-MRI applications, the CS-MMME and conventional FLASH sequences were acquired before the injection of Gd-DOTA at multiple repetition times and these sequences were repeated after the *in vivo* injection doses of 0.3 mmol/kg for four rats and 0.1 mmol/kg for other four rats. The imaging parameters of CS-MMME sequence were as follows: TR = 45 ms, 100 ms, and 250 ms, FOV = 7.5 × 7.5 × 12.8 cm^3^, Matrix: 256 × 256 × 64, Δt = 5 μs, T_ph_ ( = T_sl_) = 300 μs, ΔG_y_ ( = ΔG_z_) = 0.15 G/cm, τ_1_ = 500 μs and CS factor = 8. The imaging parameters of FLASH sequences of same resolution with reduced FOV were set as follows: TR = 10 ms, 40 ms, 160 ms, and 250 ms, TE = 3 ms (minimum), FOV = 0.2 ~ 2.4 × 8 × 8 cm^3^, Matrix: 1 ~ 12 × 128 × 128. Signal enhancements (*S_post_/S_pre_*) in kidney from two sequences with variable repetition times were compared at both injection doses for the direct comparisons on the same subject.

## Results

### Image contrast of CS-MMME with minimal susceptibility artifacts and *T*_2_* weightings

**Figure 2:**
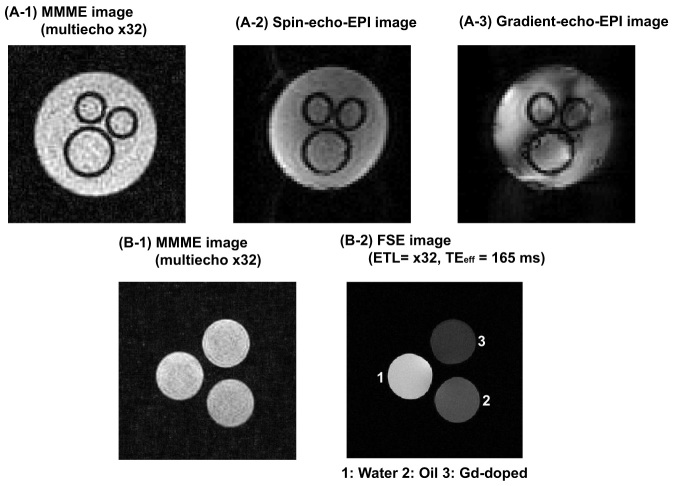
(A-1), (A-2), and (A-3) are images of the susceptibility (glass-water) phantom reconstructed by MMME (multi-echo × 32), SE-EPI, and GE-EPI sequences, respectively. (B-1) and (B-2) are images of the relaxivity (water,oil, and Gd-doped water) sample with a MMME (multi-echo × 32), and a FSE sequence with an ETL factor of 32 at an echo spacing (ES) of 11 ms (minimum) with linear *k*-space ordering, respectively.

**Figure 3:**
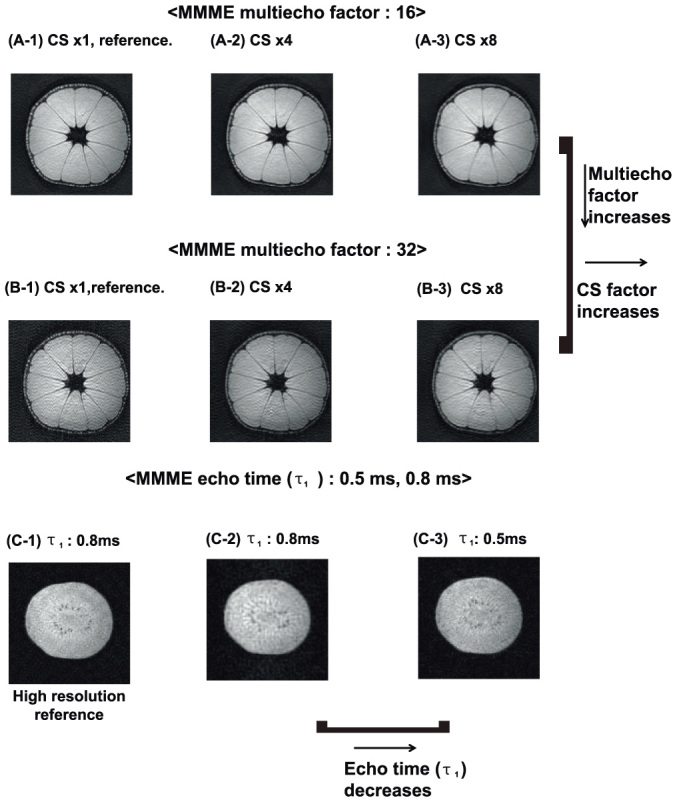
The reconstructed images of a tangerine with (A) 16 echoes and (B) 32 echoes per repetition. The images of the MMME sequence without CS are shown in (A-1) and (B-1) as references. (A-2)/(B-2) and (A-3)/(B-3) are the CS-MMME images with a CS acceleration factor of 4 and 8, respectively. Matrix size of all tangerine images were 128 × 128 × 128 with an echo time of 800 μs. (C-1) is the reconstructed high resolution kiwi image with an echo time of 800 μs, a matrix size of 256 × 256 × 64, and an acceleration factor of 8 as a reference image. (C-2) and (C-3) are the reconstructed images with echo times of 800 μs and 500 μs at a matrix size of 128 × 128 × 64 and an acceleration factor of 4, respectively.

SE-EPI and GE-EPI acquisitions for the susceptibility phantom were shown in Figures 2A-2 and 2A-3, respectively. Significant susceptibility-induced artifacts were observed in the EPI acquisitions, including ghostings and distortions due to the presence of glass tubes. In contrast, the image from the CS-MMME sequence was shown to be less prone to susceptibility artifacts with the advantage of spin-echo acquisitions, as shown in Figure 2A-1.

The image obtained from the FSE acquisition for the relaxivity phantom was shown in Figure 2B-2 with an ETL of 32 at an echo spacing of 11 ms (TE _eff_ = 165 ms). The Gd-doped sample showed the most reduced signal resulting from the shortest *T_2_* relaxation among the FSE acquisition. On the other hand, the image from the corresponding MMME sequence with a multi-echo factor of 32, showed similar intensities among the three samples. As we can see in Figure 2B-1, the non-slice-selective short MMME sequence provided accurate proton density contrast at long repetition times, independent of *T*_2_ and diffusion values, mainly due to the reduced total echo train time from non-slice-selective excitations and the echo normalization process. Considering the wide range of apparent diffusion constant (ADC) values and the *T_2_* relaxation values of the relaxivity samples, the echo amplitude normalization with respect to reference scan without phase encoding adopted in this work also appears to accurately provide spatially resolved proton density contrast for non-slice-selective 3D MMME acquisitions.

The signals for the variable-TR CS-MMME sequence from the mixture sample agarose gels with different Gd concentrations were shown in supplementary Figure 1A-1 to demonstrate the *T_1_*-weighting of the CS-MMME sequence at short repetition times. The measured *T_1_* values from the variable-TR CS-MMME sequence were then compared with those values obtained from a conventional TR-FSE (ETL = 2) sequence, yielding good agreement, and thus reflecting the spatially resolved *T_1_* contrast of the MMME sequence at short TR values (supplementary Figure 1A-2).

### Impact of CS factor, multi-echo factor, and echo time of the CS-MMME sequence

We first determined an appropriate CS acceleration factor, which balances image resolution and the required number of repetitions, by comparing the image qualities of fully acquired 3D MMME and CS-MMME (with CS acceleration factors of 4 and 8) acquisitions for a tangerine sample. The test matrix size was 128 × 128 × 128. The fully acquired 3D-MMME images with 16 and 32 echoes per MMME excitation were used as the reference images for comparison without CS acceleration. Figure 3A-1 shows an axial image of a tangerine from a 3D image acquired with an MMME multi-echo factor of 16 without CS acceleration. Figure 3B-1 shows the corresponding images with an MMME multi-echo factor of 32 without CS acceleration. The CS-MMME acquisitions with a CS factor of 4 (Figures 3A-2, 3B-2) and with a CS factor of 8 (Figures 3A-3, 3B-3) illustrated the CS induced image blurring as compared to the corresponding reference images. The qualities of image reconstructions at CS acceleration factors of 4 and 8, were morphologically consistent with that of the fully acquired 3D-MMME images, as supported by the high correlation concordance coefficient (CCC > 0.92). However, the degradations of the boundary lines of the tangerine were observed as the CS factor increases.

Next, the effects of the MMME multi-echo factor on the image quality were studied from the same datasets. For example, a smaller multi-echo factor leads to better image quality, as the poor echoes may be discarded for the encodings, but it increases the necessary number of repetitions. We also conducted experiments with MMME multi-echo factors of 16 and 32 at fixed CS acceleration factors to examine the variations in the reconstructed image quality as a function of the multi-echo factor. The image reconstructed with 16 echoes displays less background noise than the image reconstructed with 32 echoes. Experiments with a multi-echo factor of 16 at CS acceleration factors of 4 and 8 were shown in Figures 3A-2 and 3A-3. Corresponding experiments with a multi-echo factor of 32 were shown in Figures 3B-2 and 3B-3. The CS 4-fold and 8-fold accelerated images with an MMME multi-echo factor of 16 showed CCC values of 0.9909 and 0.9879, respectively. The corresponding CCC values of CS 4-fold and 8-fold accelerated images with a MMME multi-echo factor of 32 were 0.9566 and 0.9233, respectively, showing relatively lower CCC values compared with those obtained from 16 multi-echoes.

Finally, the effect of MMME echo time (τ_1_) was investigated, as it determines the required scan time of a single echo train (minimal TR), and also affects the signal of each echo. For example, the longer echo time decreases the SNR of each echo as the result of diffusion and *T_2_* relaxation. Reducing the echo time decreases the scan time per excitation and also increases the SNR of each echo. However, the required digitization time and matrix size along the frequency-encoding direction limit the minimum possible echo time. Based on these considerations, the image qualities from the CS-MMME with echo times of 800 μs and 500 μs were compared by observing the blurring of fine morphology kiwi seeds, shown in Figures 3C. The tangerine sample did not offer a significant difference in the image quality as a function of MMME echo times. First, we acquired a high-resolution image (256 × 256 × 64) with a CS acceleration factor of 8 as a reference image (Figure 3C-1). Then, an image was acquired for an echo time of 800 μs, with a smaller matrix size of 128 × 128 × 64 at a CS acceleration factor of 4, as shown in Figure 3C-2. The fine seeds were poorly defined in the lower-resolution image as compared with the corresponding higher-resolution reference image shown in Figure 3C-1. We then acquired an image using an echo time of 500 μs, a matrix size of 128 × 128 × 64, at a CS acceleration factor of 4, as shown in Figure 3C-3. For the same resolution and CS factor, the short echo-time image in Figure 3C-3 showed less blurring of the fine seeds than the longer echo-time image in Figure 3C-2, judging from the higher resolution reference image in Figure 3C-1.

### In vivo experiments with CS-MMME

**Figure 4:**
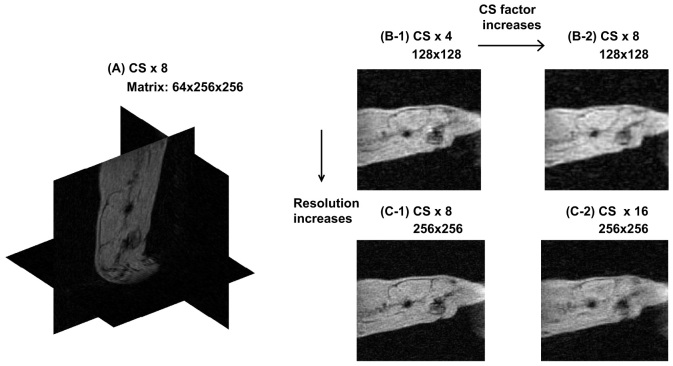
(A) shows the 3D image of a rat reconstructed with a matrix size of 256 × 256 × 64. (B-1) is the reconstructed image with an echo time of 500 μs, a matrix size of 128 × 128 × 64, a MMME factor of 32, and an acceleration factor of 4. (B-2) used the same parameters but with an acceleration factor of 8. (C-1) shows the reconstructed image with an echo time of 500 μs, a matrix size of 256 × 256 × 64, a MMME factor of 32, and an acceleration factor of 8. (C-2) used the same parameters, but with an acceleration factor of 16.

We acquired *in vivo* CS-MMME images of a rat. Sagittal slices from 3D images with matrix size of 128 × 128 × 64 at CS acceleration factors of 4 and 8 were shown Figures 4B-1 and 4B-2. Corresponding images with matrix size of 256 × 256 × 64 at CS acceleration factors of 8 and 16 were shown in Figures 4C-1 and 4C-2. All of the images were scanned using 32 echoes per excitation at a MMME echo time of 500 μs. The reconstructed higher-resolution image at a CS acceleration factor of 8, shown in Figure 4C-1, appears to provide accurate anatomical information with less blurring than the rest of other images acquired with different imaging parameters. However, the total number of repetitions in the higher-resolution image was twice (256) that of the lower-resolution image (128). Increasing the CS factor reduces the total number of excitations while inducing more blurring artifacts, further demonstrating the tradeoffs between image resolution and the total number of repetitions *in vivo*.

**Figure 5:**
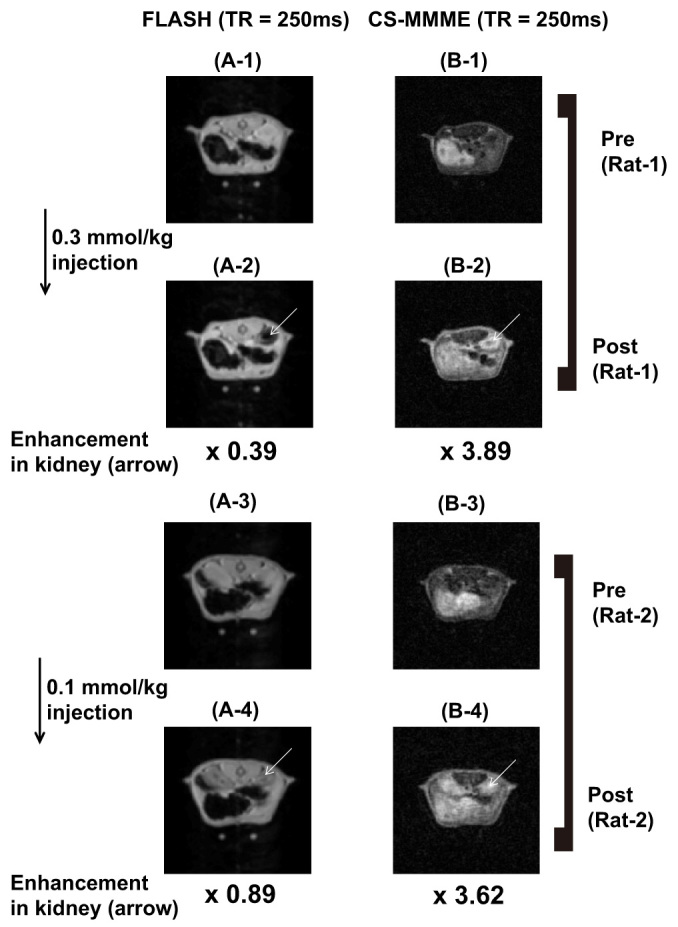
At an *in vivo* injection dose of 0.3 mmol/kg, (A-1) and (B-1) show the FLASH (TR = 250 ms), and CS-MMME (TR = 250 ms) before the injection of Gd-DTPA for the Rat-1. The corresponding post-injection images are shown in (A-2) and (B-2), respectively for Rat-1. At an *in vivo* injection dose of 0.1 mmol/kg, (A-3) and (B-3) show pre-injection images for Rat-2. (A-4) and (B-4) show corresponding post-injection images for the Rat-2. The slice direction of FLASH acquisition was aligned along the frequency encoding direction of 3D CS-MMME acquisition to match the thickness of both acquisitions.

**Figure 6:**
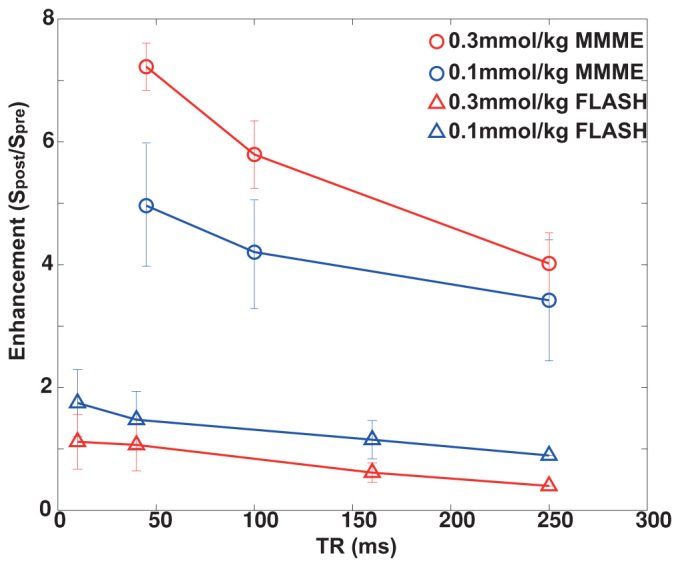
The contrast-enhanced signal ratio (*S_post_/S_pre_*) of the CS-MMME and FLASH sequences were plotted in the rat kidney at *in vivo* injection dose of 0.1 mmol/kg and 0.3 mmol/kg, as a function of repetition times (TR) at minimized echo times. Number of animals were four (*n* = 4) for each injection dose.

We finally acquired the contrast-enhanced images after the *in vivo* injections of Gd-DOTA for the CS-MMME and the corresponding FLASH images at doses of 0.3 mmol/kg and 0.1 mmol/kg for rats. Figures 5A-1 and 5B-1 showed pre-injection images of kidney region with FLASH (TR = 250 ms), and CS-MMME (TR = 250 ms) acquisitions, respectively for a rat (Rat-1). Figures 5A-2 and 5B-2 showed the respective post-injection images, which were sequentially acquired after the injections. At an injection dose of 0.3 mmol/kg, CS-MMME (TR = 250 ms) image showed a positive contrast enhancement (*S_pre_/S_post_*) of 3.89 in the kidney (indicated by the white arrow) after the injection. Negative contrast (×0.39) was observed for FLASH image with TR = 250 ms even at minimum TE (3 ms). At a lower dose of 0.1 mmol/kg for a different rat (Rat-2), CS-MMME (TR = 250 ms) image consistently showed positive contrast enhancement of 3.62 in the kidney (indicated by the white arrow) after the injection as shown in Figure 5B-4. The FLASH image showed increased but still negative enhancement (×0.89) in the kidney as shown in Figure 5A-4. Pre-injection images of this rat (Rat-2) were shown in Figures 5A-3 and 5B-3 for corresponding sequences as well. Similar comparisons were performed for other rats (*n* = 4) at different values of TR and signal enhancements (*S_post_/S_pre_*) were plotted in Figure 6 as a function of increasing TR values. Signal enhancements were seen to increase as TR decreases for both FLASH and CS-MMME sequences. CS-MMME acquisitions showed significantly improved positive contrasts at all TRs, when compared with corresponding FLASH sequences. Signal enhancements from shorter TR values (<45 ms) of CS-MMME image were not obtained due to minimally required echo train duration. However, the signal enhancement of CS-MMME image at TR = 45 ms was still larger than that of FLASH image at TR = 10 ms. The signal enhancement at higher injection dose (0.3 mmol/kg) was larger than that at low injection dose (0.1 mmol/kg) for CS-MMME acquisitions correctly reflecting the elevated *in vivo* Gd concentration, while FLASH acquisitions showed the opposite behavior of reducing contrast enhancement at higher injection dose of contrast agent due to increased T_2_* effect.

## Discussion

For the further *in vivo* applications of the proposed CS-MMME sequence, minimized T_2_* dependent weightings within a short single echo train serve as one of the benefits. More accurate 3D proton-density or *T_1_*-weighted images can be obtained for the applications, where minimization of susceptibility-induced decay is necessary. For example, varying-TR measurements with CS-MMME sequences represent potential spin-echo sequences for performing fast *T_1_* measurements with minimum T_2_* weightings as demonstrated in supplementary Figure 1. Dynamic *T_1_*-perfusion acquisitions with CS-MMME sequences are expected to suffer much weaker T_2_* effects than those in conventional gradient echo-based perfusion images. Therefore, CS-MMME sequences should provide a better estimation for contrast agent concentrations over a wider range of injection doses and repetition times, circumventing cumbersome positive contrast optimization process with conventional gradient-echo based acquisitions. As long as the minimum acquisition speed is concerned, the temporal resolution of 3D CS-MMME acquisition can potentially reach ~7 s (for a 128 × 128 × 128 matrix with an acquisition time of 128 × TR (30 ms) at a multi-echo factor of 32 and a CS factor of 4). This is significantly faster than corresponding 3D FLASH (~120 s) and 3D FSE (~190 s, ETL = 32), but similar to the acquisition speed of EPI (~10 s) sequence with same FOV and resolution in the preclinical system in this study. Assuming similar applications of CS acquisition and reconstruction to FLASH, FSE, and EPI sequences, the scan time of each sequence can be ordered as follows; 3D CS-EPI (2.5 s) < CS-MMME (7 s) < CS-FLASH (30 s) < CS-FSE (48 s) all at minimum TR and TE for same geometry^49^.

The choice of appropriate MR imaging parameters depends on the specific application of interest. This also applies to balancing the tradeoffs between accelerations and quality of the 3D CS-MMME imaging sequence. The choice of a low MMME multi-echo factor increases the SNR, but also increases the number of repetitions required to sufficiently cover 3D *k*-space, resulting in longer scan times for 3D image acquisition. Increasing the CS acceleration factor reduces the required number of repetitions but tends to induce more blurring artifacts in the reconstructed images. Reducing the MMME echo time should be beneficial for imaging applications, as short echo times minimize the diffusion and *T_2_* relaxation of each echo. However, echoes start to overlap as echo time decreases, and stronger gradients are required to separate the echoes sufficiently for proper encodings, which in turn increases the diffusion decay. Matrix sizes of 256 × 256 × 64 (128) (echo time (τ_1_): 500 μs (800 μs), CS factor: 8, total repetitions: 256) and 128 × 128 × 128 (64) (echo time (τ_1_): 800 μs (500 μs), CS factor: 4, total repetitions: 128) with 32 echoes per excitation were experimentally investigated in this work to provide morphologically correct 3D spin-echo images, including those in *in vivo* experiments with proton-density and *T_1_*-weighted contrast. It is worthwhile to note that when the echo time/separation is as short as 500 μs, you can only sample ~100 points along the frequency encoding direction with 5 μs digitization time and that's why we set 64 points along the frequency encoding direction with shortest echo time of 500 μs. If necessary, either digitization time can be decreased or echo time/separation can be increased to obtain higher matrix size along the frequency encoding directions for the specific applications. It also should be noted that multi-echo accelerations induced amplitude inhomogeneities across the sample, and image blurring were introduced with CS accelerations. Future optimizations may be focused on further suppressing these artifacts, balancing between both multi-echo and CS accelerations.

The clinical applications of the CS-MMME sequence are beyond the scope of this work, but they are worth mentioning. The restricted maximum strength (~4 G/cm) and the slew rate (~200 T/m/s) of clinical gradient systems will pose limitations on the key parameters of the CS-MMME sequence. For example, to achieve an in-plane resolution of 1 mm (FOV = 26 cm, matrix = 256 × 256) in a 3 T clinical scanner, the required strengths of the constant frequency-encoding gradient, the maximum phase-encoding gradient, and the minimum echo time will be 1.8 G/cm, 3.8 G/cm, and 1.5 ms, respectively, as the minimum blip time of the phase-encoding gradient tends to increase from the reduced slew rate of clinical systems. This will lead to an increased minimum echo time of the sequence, increasing the minimum total echo train time to 65 ms from 30 ms for the preclinical scanner with a higher slew rate (~3000 T/m/s). For the regional investigation, saturating pre-pulse may be included before the 5 RF pulse echo train to localize the region of interest as demonstrated in previous study^19^ or each pulse can be made to be slice-selective at the cost of the lengthened echo time/separation.

We demonstrated the feasibility of CS-MMME sequences for acquiring high-resolution 3D images requiring far fewer excitations on various phantoms and *in vivo* animals with proton-density and *T_1_*-weighted contrast. The direct benefits are two folds. First, the increased pulse to spin-echo ratio of the CS-MMME sequence (32 echoes/5 RF pulses = 6.4) enables accelerated proton-density or *T_1_*-weightings with reduced RF irradiations at the speed comparable to that of EPI. Second, the CS-MMME sequence with minimized total echo train time (30–50 ms) reduces TE dependent signal decays from T_2_* relaxation, even at a large multi-echo factor (×32) per a repetition. On the other hand, it should be noted that proper echo normalization is necessary from multi-echo accelerations. Image blurring is induced as high CS acceleration is employed depending on the anatomy of interest, while this may help further suppressing inhomogeneous multi-echo artifacts with further investigations.

In conclusion, by simultaneously acquiring 128 ~ 256 effective phase-encoding steps in a single repetition, while retaining in-plane image resolution of 200 μm, the CS-MMME sequence opens up the possibility of significantly accelerated 3D *T_1_*-weighted spin-echo-train acquisitions for positively contrasted-enhanced *in vivo* MR applications, where minimization of susceptibility-induced decay is necessary both at high temporal resolution and minimized RF irradiations.

## Author Contributions

S.H.H. and F.H.C. developed pulse sequences and performed experiments. Y.K.S. performed *in vivo* experiments. J.P. and Y.Q.S. wrote simulation code. H.J.C. conceived the experiment. S.H.H., G.G.C., J.K.K., Y.R.K. and H.J.C. designed experiments and wrote the manuscript. All authors reviewed the manuscript.

## Supplementary Material

Supplementary Information Supplementary information
