# Supplementary material for: Ultrafast 3D spin-echo acquisition improves Gadolinium-enhanced MRI signal contrast enhancement
Source: Sci Rep. 2014 May 27;4:5061. doi: 10.1038/srep05061 (PMC4034007; doi:10.1038/srep05061)

Supplementary Information

# **Ultrafast 3D spin-echo acquisition improves Gadolinium-enhanced MRI signal contrast enhancement**

*S.H. Han^a^, F.H. Cho^a^, Y.K Song^a^ ,J. Paulsen^b^, Y.Q.Song^b^, Y.R.Kim^c^, J.K.Kim^d^, G. Cho^e, *^, and H. Cho^a,*^*

**Supplementary Figure 1.** (A-1) shows the signal at variable TR with 0.05% agarose gel with different relaxation times from various Gd-doping conditions. The *T_1_*-fittings are shown in by solid lines. (A-2) compares the fitted *T_1_* values from the variable TR CS-MMME sequence with those from conventional variable TR (TR-FSE, ETL=2) measurements.


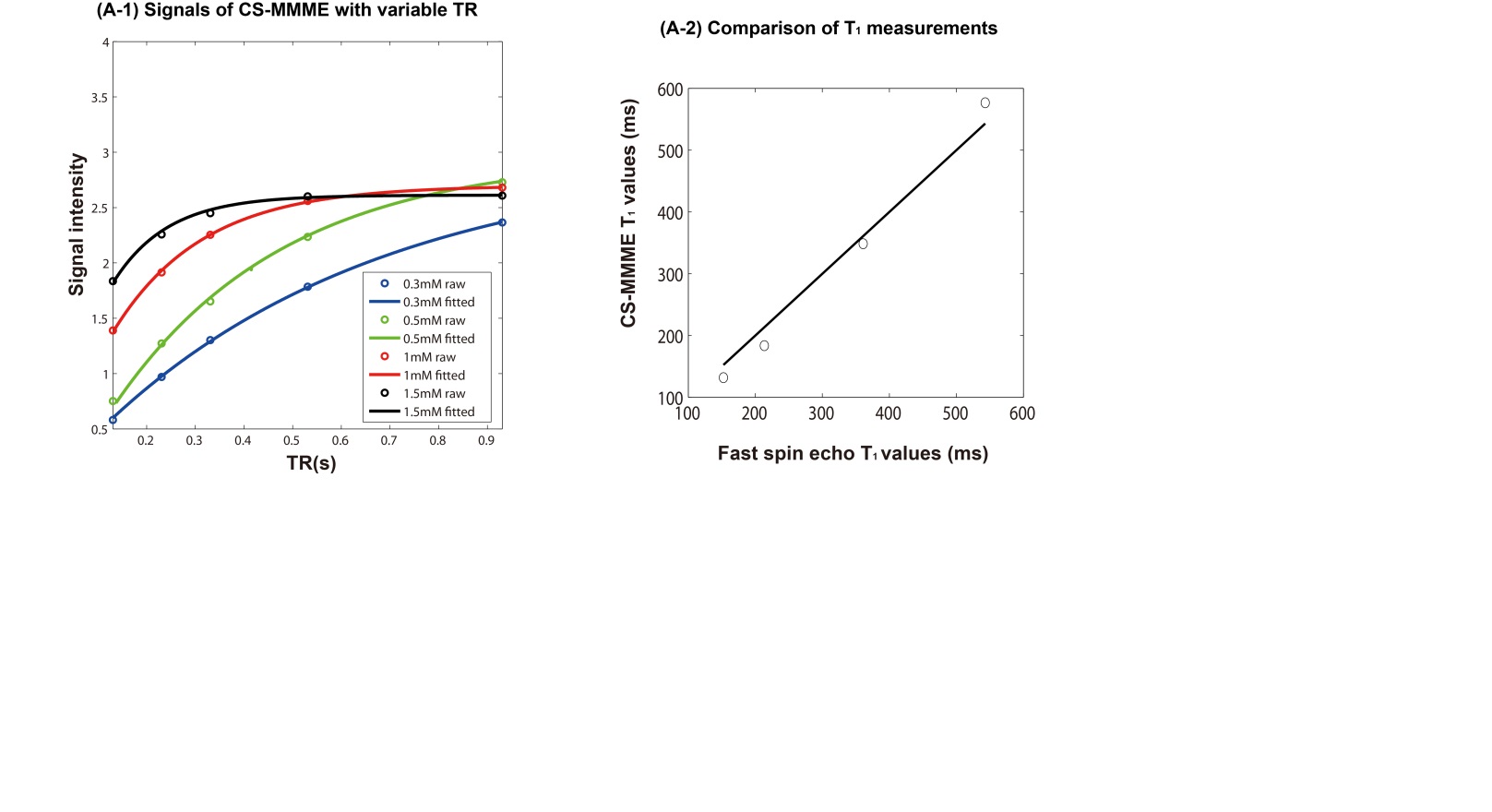

Supplement: Supplementary Information [file srep05061-s1.doc]
